# The impact of circulating tumor DNA on the prognosis of liver cancer and its predictive value: a meta analysis

**DOI:** 10.3389/fgene.2026.1767755

**Published:** 2026-02-12

**Authors:** Bing Wu, Shuhui Ke, Lingling Zhu, Rongrong Dong, Jinqian Luan

**Affiliations:** 1 Department of Laboratory, Jiaojiang Maternal and Child Health Hospital, Taizhou, China; 2 Department of Gynecology, Jiaojiang Maternal and Child Health Hospital, Taizhou, China; 3 Department of Obstetrics, Jiaojiang Maternal and Child Health Hospital, Taizhou, China

**Keywords:** circulating tumor DNA, hepatocellular carcinoma, liquid biopsy, meta-analysis, prognosis, recurrence, survival prediction

## Abstract

**Background:**

ctDNA is a promising biomarker in oncology. However, its prognostic and predictive value in HCC remains underexplored. This meta-analysis aims to evaluate the prognostic impact of ctDNA in HCC and its predictive value for recurrence.

**Methods:**

A systematic review and meta-analysis were performed following PRISMA guidelines. PubMed, Embase, Web of Science, and CNKI were searched up to 1 June 2025, for studies assessing ctDNA in HCC patients with reported survival outcomes or predictive accuracy. Studies reporting hazard ratios for overall or disease-free survival, or AUCs for prediction, were included. Two reviewers independently screened studies and assessed quality using the Newcastle-Ottawa Scale (NOS). Meta-analyses used random- or fixed-effects models depending on heterogeneity, with sensitivity analyses performed to assess robustness.

**Results:**

A total of 219 records were screened from PubMed, Embase, Web of Science, and CNKI, and 8 studies comprising 1,907 patients were included. ctDNA positivity was significantly associated with poorer OS, with a pooled HR of 2.34 (95% CI 1.96–2.78; p < 0.0001). Moderate heterogeneity was observed (I^2^ = 32.2%). Sensitivity analyses confirmed the robustness of this finding. Two studies assessed the predictive value of ctDNA for RFS, yielding a pooled AUC of 0.66 (95% CI 0.47–0.86; I^2^ = 65.7%). Discriminative accuracy was higher when ctDNA was detected postoperatively (AUC range: 0.57–0.77), suggesting its potential role in identifying minimal residual disease.

**Conclusion:**

ctDNA is associated with adverse prognosis in HCC and may offer moderate predictive accuracy for recurrence. Standardized protocols for sampling and analysis are required to facilitate broader clinical translation.

## Introduction

Hepatocellular carcinoma (HCC) is one of the most prevalent malignancies worldwide and represents a major cause of cancer-related mortality (Chen et al., 2020). Its global burden continues to rise, particularly in regions with high prevalence of chronic hepatitis B and C infection, as well as among populations affected by alcohol-related liver disease and non-alcoholic fatty liver disease (Court et al., 2018). Despite advances in surgical resection, liver transplantation, and systemic therapies, the prognosis for patients with HCC remains poor, characterized by high recurrence rates and limited overall survival, especially in advanced stages (El-Mezayen et al., 2022).

Accurate risk stratification and early recurrence detection are critical for improving clinical outcomes in HCC (Jiang et al., 2024). Conventional surveillance strategies, including imaging and serum biomarkers such as alpha-fetoprotein, often lack the sensitivity and specificity required for timely and individualized management (El-Mezayen et al., 2022). These limitations have prompted the search for novel, dynamic biomarkers capable of capturing the molecular and biological complexity of HCC throughout the disease course (Jiang et al., 2024).

Circulating tumor DNA (ctDNA), comprising fragments of tumor-derived genetic material released into the bloodstream, has emerged as a promising non-invasive biomarker (Alemayehu et al., 2024a). In HCC, growing evidence suggests that the presence and quantitative characteristics of ctDNA may be associated with clinical outcomes, including disease-free survival and overall survival. However, existing studies vary widely in methodology, detection techniques, and clinical endpoints, leading to inconsistent findings and uncertainty regarding the clinical utility of ctDNA in routine practice (Alemayehu et al., 2024b).

In this study, we conducted a comprehensive meta-analysis to evaluate the prognostic and predictive value of ctDNA in patients with HCC. By systematically synthesizing the current evidence, we aim to clarify the role of ctDNA in forecasting clinical outcomes and to support its potential integration into the prognostic framework of HCC management.

## Methods

### Search strategy

We conducted a systematic review and meta-analysis in accordance with PRISMA guidelines. A comprehensive search was conducted in PubMed, Embase, Web of Science, and CNKI from inception to 1 June 2025, using combinations of the following terms: “hepatocellular carcinoma”, “circulating tumor DNA”, “ctDNA”, “prognosis”, “survival”, “recurrence”, and “liquid biopsy”. No language restrictions were applied. Additional studies were identified by screening reference lists of relevant articles and reviews. A complete list of search terms and strategies is provided in Supplementary Table S1.

### Selection criteria

Studies were included if they met the following criteria (Chen et al., 2020): patients were diagnosed with hepatocellular carcinoma based on pathological or radiological criteria (Court et al., 2018); ctDNA was evaluated after curative-intent therapy in peripheral blood using any validated molecular method. A sample was considered ctDNA-positive if at least one mutation was detected in ctDNA with the matched tumor (El-Mezayen et al., 2022); the study reported hazard ratios (HRs) with corresponding 95% confidence intervals (CIs) for overall survival (OS) and/or disease-free survival (DFS), or provided sufficient data to estimate HRs; and/or (Jiang et al., 2024) the study assessed the discriminatory ability of ctDNA for predicting survival outcomes using the time-dependent area under the curve (AUC). Case reports, reviews, conference abstracts without sufficient data, and studies with overlapping patient populations were excluded.

### Data extraction and quality assessment

Two reviewers independently screened titles, abstracts, and full-text articles for eligibility. Discrepancies were resolved through discussion or consultation with a third reviewer. When multiple models were presented, the most adjusted HRs were prioritized. Study quality was assessed using the Newcastle-Ottawa Scale (NOS) for cohort studies.

### Outcomes

The primary outcome was the association between ctDNA status (positive vs. negative or high vs. low levels) and survival outcomes, expressed as HRs for overall survival and disease-free survival. The secondary outcome was the predictive accuracy of ctDNA for recurrence-free survival, assessed by time-dependent AUCs.

### Statistical analysis

Meta-analysis was performed using R software (version 4.3.3) with the “meta” and “metafor” packages. For predicted outcomes, the area under the curve (AUC) values were used, while for dichotomous outcomes, HR were calculated. The results were expressed as odds ratios with 95% confidence intervals (CIs). Heterogeneity was assessed using the I^2^ statistic, with a random-effects model applied if I^2^ ≥ 50%, and a fixed-effect model used if I^2^ < 50%. Sensitivity analysis was performed to assess the robustness of the results.

## Results

### Study selection and characteristics

**FIGURE 1:**
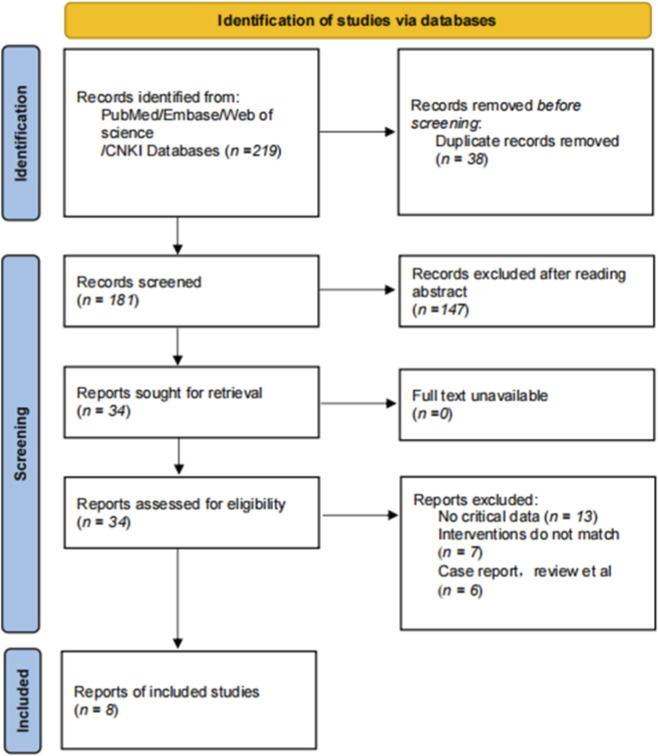
Flow chart.

**FIGURE 2:**
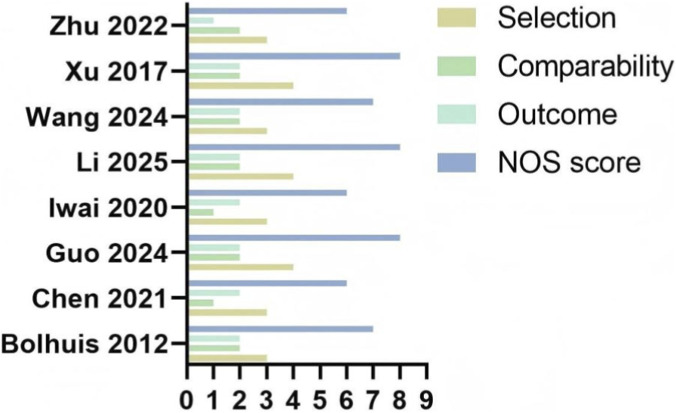
Summary of risk of bias.

A total of 219 records were retrieved from PubMed, Embase, Web of Science, and China National Knowledge Infrastructure (CNKI). After removing 38 duplicates, we screened 181 unique articles by title and abstract, excluding 147 for irrelevance. We assessed the full texts of the remaining 34 articles and identified 8 eligible studies (Bolhuis et al., 2021; Chen and Kaiwen, 2021; Guo et al., 2024; Iwai et al., 2020; Li et al., 2025; Wang et al., 2024a; Xu et al., 2017; Zhu et al., 2022), comprising a combined total of 1,907 patients (Figure 1). Quality assessment using the Newcastle-Ottawa Scale (NOS) indicated moderate to high methodological quality across all studies, with a median score of 7 (Figure 2).

Baseline characteristics of the included cohorts are presented in Table 1. The pooled population was predominantly male (78.3%, 1,493/1,907), with a median age of 55 years (range: 15–85 years). The included studies varied considerably in sample size, ranging from 23 to 1,098 participants (Table 1).

**TABLE 1:** Baseline characteristics.

First author	Publication year	Number of cases	Age (year)	Gender		ctDNA calculating methods	Countries /Regions
				Male	Female		
Bolhuis et al. (2021)	2021	23	63 (54–76)	8	15	ddPCR	Netherlands
Chen and Kaiwen (2021)	2021	208	53.91 ± 8.24	162	46	ddPCR	China
Guo et al. (2024)	2024	293	51 (30–81)	246	47	qPCR	China
Iwai et al. (2020)	2020	41	69 (18–85)	25	16	qPCR	Japan
Li et al. (2025)	2025	126	Non-recurrence group 49.42 ± 10.07 Recurrence group 52.31 ± 10.77	96	30	ddPCR	China
Wang et al. (2024a)	2024	67	≥60 27cases; <60 40cases	39	28	qPCR	China
Xu et al. (2017)	2017	1,098	55 (15–81)	905	130	ddPCR	China
Zhu et al. (2022)	2022	41	>50 34cases; <50 7cases	35	6	ddPCR	China

### Prognostic impact of ctDNA on survival

**FIGURE 3:**
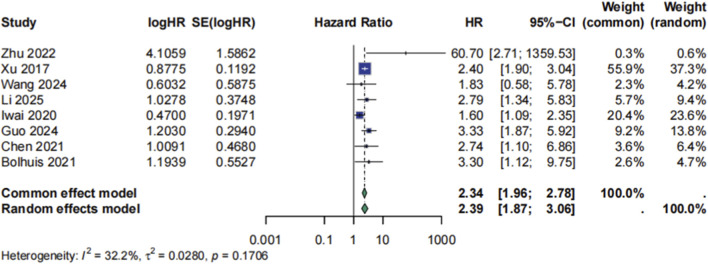
Meta analysis for HR of prognosis.

**FIGURE 4:**
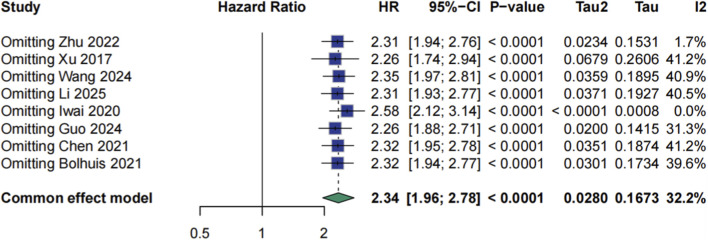
Sensitive analysis for HR of prognosis.

Meta-analysis using a random-effects model demonstrated a significant association between ctDNA positivity and worse overall survival (OS), with a pooled hazard ratio (HR) of 2.34 (95% CI: 1.96–2.78, p < 0.0001; Figure 3). Moderate heterogeneity was observed (I^2^ = 32.2%, τ^2^ = 0.0280, p = 0.17). Sensitivity analyses excluding each study in turn yielded consistent results, with HRs ranging from 2.26 to 2.58, indicating the robustness of the association (Figure 4).

### Predictive value for recurrence-free survival (RFS)

**FIGURE 5:**
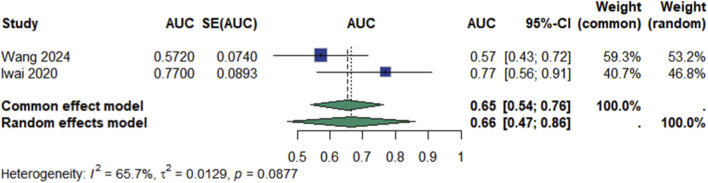
Meta analysis of Recurrence-Free Survival.

Two studies (Iwai et al., 2020; Wang et al., 2024b) reported on the predictive accuracy of ctDNA for RFS. The pooled area under the curve (AUC) was 0.66 (95% CI: 0.47–0.86), with substantial heterogeneity (I^2^ = 65.7%, p = 0.09; Figure 5). Notably, the discriminative ability was higher in Iwai 2020 (AUC: 0.77 [0.56–0.91]) than in Wang 2024 (AUC: 0.57 [0.43–0.72]), which may reflect differences in ctDNA sampling timepoints or methodological approaches.

## Discussion

This meta-analysis underscores the significant prognostic relevance of ctDNA in patients with HCC (Huang et al., 2022). The consistent association between ctDNA positivity and poor OS across studies suggests that ctDNA is not merely a reflection of tumor burden but a biologically meaningful marker of aggressive disease phenotypes (Zhang et al., 2021). It likely integrates multiple dimensions of tumor biology, including proliferative potential, vascular invasion, and metastatic propensity (Aalami et al., 2024). Notably, the prognostic value of ctDNA is not unique to HCC. A recent meta-analysis in pancreatic malignancies also reported that ctDNA-positive status was significantly associated with worse overall survival (HR = 2.00), reinforcing the role of ctDNA as a robust, pan-cancer prognostic biomarker across different solid tumors (Arayici et al., 2024).

The biological rationale supporting the prognostic role of ctDNA lies in its origin and dynamics. ctDNA is released into circulation during cellular apoptosis, necrosis, or active secretion by tumor cells, with its abundance influenced by factors such as tumor size, vascularity, immune clearance, and cellular turnover (Tan et al., 2021). In HCC, where tumors are often heterogeneous and vascularized, ctDNA may serve as a proxy for intratumoral heterogeneity and the presence of aggressive subclones (Cheng et al., 2019). The observed association with OS may therefore reflect an underlying biological shift toward systemic dissemination and resistance to host defenses or therapy (Cui et al., 2020).

The moderate heterogeneity observed among included studies is likely attributable to differences in detection platforms, patient populations, and definitions of ctDNA positivity (Chandrapalan et al., 2022). Some studies utilized PCR-based methods, which target specific mutations with high sensitivity, while others applied broader sequencing techniques that allow for comprehensive mutation profiling but with variable analytical depth (Li et al., 2022). Despite these methodological differences, sensitivity analyses confirmed the robustness of the association with OS, suggesting that the prognostic value of ctDNA remains consistent regardless of assay modality (Oussalah et al., 2018).

In evaluating the predictive performance for RFS, the pooled AUC indicated only modest discriminative accuracy (Liu et al., 2022). This may reflect, in part, methodological heterogeneity among the included studies, particularly regarding the timing and frequency of ctDNA sampling. Studies that performed ctDNA detection during the postoperative or surveillance phase generally reported higher AUCs, suggesting a more effective role for ctDNA in identifying minimal residual disease. Mechanistically, this aligns with the concept that residual malignant clones shed fragmented DNA into the circulation before they are radiologically detectable. ctDNA may thus serve as a leading indicator of molecular relapse, offering a window for earlier intervention (Lumkul et al., 2024). In contrast, pre-treatment ctDNA levels may reflect bulk tumor burden rather than residual disease potential, limiting their utility in forecasting recurrence. These observations underscore the importance of temporal context in ctDNA interpretation and point to the need for standardized protocols regarding assay timing and clinical thresholds.

From a translational perspective, ctDNA holds several theoretical and practical advantages over conventional surveillance tools in hepatocellular carcinoma. Biologically, ctDNA reflects tumor-derived genetic and epigenetic alterations, such as mutations in TP53, TERT promoter, or CTNNB1, as well as methylation changes and fragmentomic signatures. These features allow for a more nuanced understanding of tumor behavior, clonal evolution, and response dynamics (Lumkul et al., 2024). Unlike static biomarkers such as AFP or imaging modalities that rely on structural changes, ctDNA enables real-time monitoring of tumor kinetics and treatment efficacy, potentially capturing molecular resistance before clinical progression (Jin et al., 2019). Furthermore, in patients with impaired hepatic function or elevated bleeding risk, where invasive biopsy is contraindicated, ctDNA offers a safe and repeatable alternative for tumor genotyping and longitudinal assessment. The ability to track tumor-specific molecular profiles through serial blood sampling may ultimately support a shift toward more personalized, adaptive management strategies in HCC (Lumkul et al., 2024). However, the implementation of ctDNA-based monitoring in routine practice requires rigorous validation of its sensitivity, specificity, and cost-effectiveness in prospective studies.

Despite these promising implications, this meta-analysis has several limitations. First, the number of included studies was limited, and their predominantly retrospective design may introduce bias and confounding. Additionally, substantial variability in ctDNA detection methods, thresholds, and sampling timepoints, coupled with the frequent lack of key methodological details such as the blood volume used for cfDNA isolation, may compromise the comparability and sensitivity of ctDNA assessments across studies. Moreover, most studies did not adjust for clinical variables such as tumor stage or treatment type, limiting interpretation of independent prognostic value. Furthermore, the assessment of ctDNA’s predictive accuracy for recurrence was based on only two studies with significant heterogeneity, which precludes strong conclusions in this regard. Finally, the generalizability of our findings may be constrained by the geographical concentration of the included patient cohorts, which were primarily from Asian and European populations. Therefore, future research is needed to validate the prognostic value of ctDNA in broader geographical and ethnic cohorts.

In conclusion, this study supports ctDNA as a promising biomarker for prognostication and recurrence prediction in HCC. Its detection correlates with worse survival outcomes and shows potential for early identification of molecular relapse. However, broader validation in prospective, well-annotated cohorts is necessary. Standardized workflows, defined reporting frameworks, and integration with imaging and clinical parameters will be key to realizing the clinical utility of ctDNA in the management of HCC.

## Data Availability

The original contributions presented in the study are included in the article/Supplementary Material, further inquiries can be directed to the corresponding author.
