# Supplementary material for: The impact of circulating tumor DNA on the prognosis of liver cancer and its predictive value: a meta analysis
Source: Front Genet. 2026 Feb 12;17:1767755. doi: 10.3389/fgene.2026.1767755 (PMC12935317; doi:10.3389/fgene.2026.1767755)
Supplement: Supplementary file 1 [file Table1.docx]

Supplementary Table 1

| Database | Retrieval strategy |
| --- | --- |
| Pubmed | ("Hepatocellular Carcinoma"[Mesh] OR "Liver Neoplasms"[Mesh] OR hepatocellular carcinoma OR liver cancer OR liver tumor OR HCC) AND ("Circulating Tumor DNA"[Mesh] OR ctDNA OR "circulating tumor DNA" OR "circulating tumor DNA" OR "liquid biopsy") AND (prognosis OR prognostic OR survival OR "overall survival" OR "disease-free survival" OR "recurrence-free survival" OR AUC OR "area under the curve") |
| Embase | ('hepatocellular carcinoma'/exp OR 'liver cancer':ti,ab,kw OR 'liver tumor':ti,ab,kw OR 'hcc':ti,ab,kw) AND ('circulating tumor dna'/exp OR 'ctdna':ti,ab,kw OR 'circulating tumor dna':ti,ab,kw OR 'liquid biopsy':ti,ab,kw) AND ('prognosis'/exp OR 'prognostic':ti,ab,kw OR 'survival':ti,ab,kw OR 'overall survival':ti,ab,kw OR 'disease free survival':ti,ab,kw OR 'area under the curve':ti,ab,kw OR auc:ti,ab,kw) AND 'clinical article'/de |
| Web of Science | TS = (("hepatocellular carcinoma" OR "liver cancer" OR "liver tumor" OR HCC)  AND ("circulating tumor DNA" OR "circulating tumor DNA" OR ctDNA OR "liquid biopsy")  AND (prognosis OR prognostic OR survival OR "overall survival" OR "disease-free survival" OR "recurrence-free survival" OR AUC OR "area under the curve") AND "clinical trial" ) |
| CNKI | SU = ('liver cancer' + 'hepatocellular carcinoma' + 'HCC') AND SU = ('circulating tumor DNA' + 'ctDNA' + 'liquid biopsy') AND SU = ('prognosis' + 'survival' + 'overall survival') |
